# Sequence-based prediction of protein binding mode landscapes

**DOI:** 10.1371/journal.pcbi.1007864

**Published:** 2020-05-26

**Authors:** Attila Horvath, Marton Miskei, Viktor Ambrus, Michele Vendruscolo, Monika Fuxreiter

**Affiliations:** 1 MTA-DE Laboratory of Protein Dynamics, Department of Biochemistry and Molecular Biology, University of Debrecen, Debrecen, Hungary; 2 The John Curtin School of Medical Research, The Australian National University, Canberra, Australia; 3 Centre for Misfolding Diseases, Department of Chemistry, University of Cambridge, Cambridge, United Kingdom; National Cancer Institute, United States of America and Tel Aviv University, Israel, UNITED STATES

## Abstract

Interactions between disordered proteins involve a wide range of changes in the structure and dynamics of the partners involved. These changes can be classified in terms of binding modes, which include disorder-to-order (DO) transitions, when proteins fold upon binding, as well as disorder-to-disorder (DD) transitions, when the conformational heterogeneity is maintained in the bound states. Furthermore, systematic studies of these interactions are revealing that proteins may exhibit different binding modes with different partners. Proteins that exhibit this context-dependent binding can be referred to as fuzzy proteins. Here we investigate amino acid code for fuzzy binding in terms of the entropy of the probability distribution of transitions towards decreasing order. We implement these entropy calculations into the FuzPred (http://protdyn-fuzpred.org) algorithm to predict the range of context-dependent binding modes of proteins from their amino acid sequences. As we illustrate through a variety of examples, this method identifies those binding sites that are sensitive to the cellular context or post-translational modifications, and may serve as regulatory points of cellular pathways.

## Author summary

Great advances have been made in the last several decades in deciphering how the behavior of proteins is encoded in their amino acid sequences. A variety of sequence-based prediction methods have been developed to estimate a wide range of properties of proteins, including secondary structure propensity, native state structures, preference for being disordered and tendency to aggregate. Much less is known, however, about the rules that regulate the conformational changes of proteins upon binding. In particular, many proteins change their binding modes upon interacting with different partners, or as a consequence of post-translational modifications or changes in the cellular milieu. Here we address the problem of how amino acid sequences can encode different binding modes depending on their binding partners, and describe the FuzPred method of predicting context-dependent binding modes.

## Introduction

With the advent of fast sequencing methods there has been an explosion in the number of proteins of known amino acid sequence. As the number of proteins whose sequences have been determined currently vastly exceeds that of proteins with known structures, especially in functional forms, one can exploit this asymmetry of information to develop sequence-based predictors of protein conformational behaviour. Great advances have been made in this area, with several methods introduced in the last two decades [1–4].

Another major recent advance in molecular biology has been the discovery of disordered proteins, which do not fold into well-defined three-dimensional structures but remain conformationally heterogeneous in their native states [5, 6]. This discovery has further promoted the development of sequence-based prediction methods to facilitate the study of the properties of these proteins. While we have currently reached a good consensus about the prediction of the degree of disorder of these proteins in their monomeric states [7, 8], there is still work to do to predict what happens upon binding [9]. Disordered regions function in many cases via gaining a well-defined structure upon interacting with their partners [10]. It has also been suggested that versatile target selectivity via templated folding is enabled by heterogeneous contacts at the transition state [11]. Experimental data demonstrate that disorder can persist [12, 13], and even increase upon interactions [14]. More recently it has also been realised that the presence of multiple modes, or fuzziness, in protein interactions is also required for liquid-liquid phase separation [15, 16].

**Fig 1:**
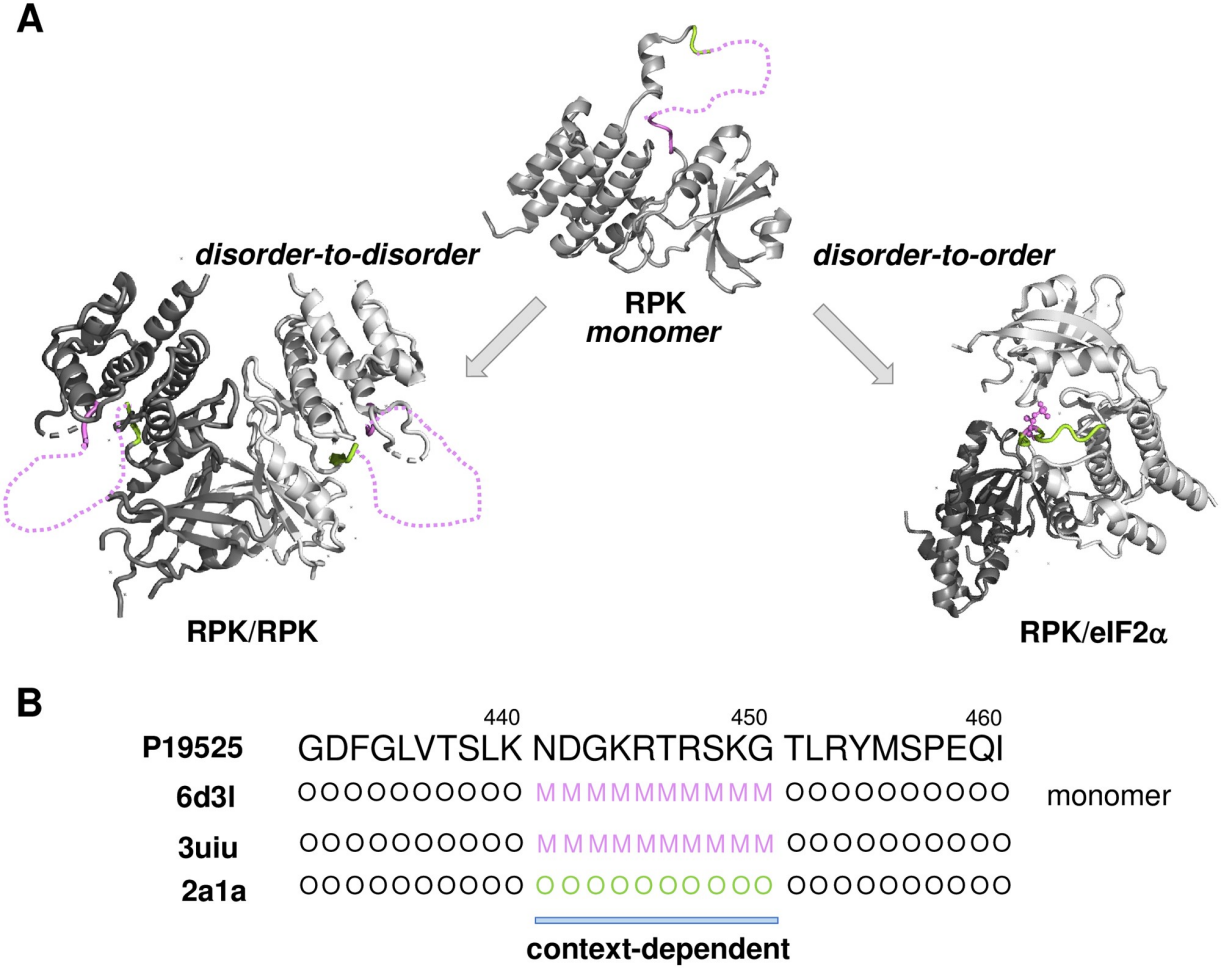
Illustration and assignment of binding modes. **(A) Binding modes considered in this work.** Binding modes are shown for the interferon-induced, double-stranded RNA-activated protein kinase (RPK). The activation segment (residues 440–450) is not visible in the crystal structure of the monomeric form (PDB: 6d3l [52]), and remains disordered in the dimeric form (PDB: 3uiu, 6d3k). This binding mode represents a disorder-to-disorder transition. Interactions with eukaryotic initiation factor 2 (eIF2), however, triggers folding of the activation segment to mediate inter-molecular contacts (PDB:2a1a [53]), which process is coupled to auto-phosphorylation of Thr446. The RPK binding to eIF2 is classified as a disorder-to-order transition. **(B) Assignment of context-dependent binding.** Structures corresponding to the same sequence (P19525, residues 440–460) were collected in the monomeric and complex forms. Residues were observed (O) in the crystal structures were assigned as 'ordered', missing residues (M) were assigned as disordered. 'Context-dependent' residues (blue bar) were disordered in the monomeric form, but were represented both in ordered and disordered forms in different complexes. 'Disorder-to-order' residues were disordered in the monomeric structure and ordered (O) in all complexes; whereas 'disorder-to-disorder' residues also remained to be disordered (M) in all bound state structures.

In addition, certain proteins have evolved the ability to adopt different binding modes depending on their binding partners, which has been termed context-dependent binding (Fig 1). Disordered regions, in particular, often act as interaction hubs [17], and different partners may require different modes of binding. To offer an example, the N-terminal region of glycogen synthase kinase-3 (GSK3) can establish a well-defined structure and interactions with partners in the insulin pathway, while remaining dynamic and exhibiting a variety of weak binding modes with partners in the Wnt pathway [18]. Interconversion between ordered and dynamic interactions can also take place after the complexes are formed, and could be regulated by post-translational modifications [19]. Variations between binding modes may activate different cellular pathways. For instance, the active state of β2-adrenergic receptor (ADRB2) is not fully stabilized by high-affinity agonists, which enables allosteric regulation by G-proteins [20], so that switching between different binding modes in the bound form regulates multiple signalling pathways via a dynamical coupling to the G-protein interface [21].

How can different binding modes be encoded in the same sequence? While a repertoire of methods for predicting the degree of disorder in the monomeric state of proteins are available [7, 8], we have a more limited knowledge of the conformational transitions that occur upon binding. In particular, we would like to increase our understanding how binding modes of a protein, or a protein region, can be modulated according to the cellular context. Recently, we have demonstrated that a wide range of binding modes of proteins are encoded in their amino acid sequences and can be predicted without specific information about their partners using the FuzPred method [22]. Here we show that it is possible to use this method not only to identify the most likely binding mode, but also to evaluate the tendency to adopt alternative binding modes.

## Results

### Binding modes of disordered proteins

In this work, we considered three types of binding modes for interactions of disordered regions (Fig 1). Disorder-to-order (DO) transitions take place when disordered regions fold upon binding into well-defined conformations. Disorder-to-disorder (DD) transition happen instead when disordered regions still exhibit conformational heterogeneity in the bound states, either by folding into alternatively conformations [23] or fluctuating while interacting with their partners [24]. Context-dependent (CD) transitions can be observed when disorder-to-order or disorder-to-disorder transitions are established in different complexes (Fig 1A). We will also refer this binding mode as conditional ordering, reflecting conditional folding with specific partners or conditions. Our work is aimed to distinguish context-dependent regions (CDRs) with a multiplicity of binding modes from disorder-to-order regions (DORs) and disorder-to-disorder regions (DDRs), which were observed only in one state (either ordered or disordered) in their complexes.

Disordered protein regions representing the three types of binding modes interactions have been collected from the Protein Databank (PDB) based on missing electron density in the corresponding crystal structures (Fig 1). DORs were defined as disordered regions in the monomeric state, while gained a well-defined structure in all representative complexes (Methods, Fig 1, S1 Table). In contrast, DDRs were identified as those regions that remained disordered in the bound states (Methods, Fig 1, S1 Table). CDRs were defined as those regions that were disordered in the monomeric state, while being observed in both structured and disordered states in different complexes (Methods, Fig 1B, S1 Table). In this study, only regions with at least one residue mediating inter-molecular interactions in the bound form were included (Methods). Structural evidence in PDB, however, does not indicate whether regions undergoing disorder-to-disorder transitions do contribute to intermolecular interactions. Thus, we have been assembled fuzzy, disordered binding regions from the Fuzzy Complexes Database (FuzDB, http://protdyn-database.org) [25], which also informs on the contributions to binding (Methods, S1 Table). The possible mechanisms how fuzzy regions impact specific partner recognition have been reviewed elsewhere [9, 26, 27].

### Probabilities of the binding modes of disordered regions

The characterisation of disorder in the bound states presents a challenge for disorder prediction methods, which have been developed for predicting disorder in the free state of proteins. Previously, we have applied different disorder prediction algorithms (IUPred [28], Dynamine [29], Disopred3 [30] and Espritz NMR [31]) using different versions and thresholds to identify regions that remain disordered in the bound states [22], finding that these methods could not be applied to robustly identify DDRs from the amino acid sequences. For reference, Espritz NMR [31] exhibited the highest performance out of these approaches, with a segmental overlap value [32] of SOV = 47.4% [22].

Instead of using the degree of disorder in the free state, we found that local biases in the sequence composition of the binding regions as compared to their flanking regions can distinguish between disorder-to-order and disorder-to-disorder regions [22], and the discrimination is robust using different flanking window sizes and different disorder prediction algorithms [22]. To implement these observations into the FuzPred prediction method, we determined the difference in disorder scores (Δ*ID*_*R*,*Fl*_) by Espritz NMR (S1 Text), and computed the differences in amino acid composition (Δ*A*_*R*,*Fl*_) and hydrophobicity (Δ*H*_*R*,*Fl*_) of the binding sites with respect to their 20-residue flanking segments (S1 Text). We demonstrated that these biases in disorder, composition and hydrophobicity significantly discriminate between DORs and DDRs [22].

In the FuzPred method, we characterise the binding modes of disordered regions by the probabilities of their transitions upon binding towards increasing (*p*_*DO*_) and or decreasing (*p*_*DD*_) order. Such probabilities were derived from a binary logistic regression model as [22]

$$p_{DO\;}\left(R\right)\;=\;\frac{exp\;S_{F}\left(R\right)}{1\;+\;exp\;S_{F}\left(R\right)}$$ (1)

where *p*_*DO*_*(R)* is the probability of disorder-to-order transition, R is the interacting region, and *S*_*F*_*(R)* is the scoring function

$$S_{F}(R)=\lambda_{1}*\Delta {ID}_{R,FL}+\lambda_{2}*\Delta A_{R,Fl}+\lambda_{3}*\Delta H_{R,Fl}+\gamma$$ (2)

where the three variables are the local biases in disorder propensity (Δ*ID*_*R*,*Fl*_), amino acid composition (Δ*A*_*R*,*Fl*_) and hydrophobicity (Δ*H*_*R*,*Fl*_) of region R as compared to the flanking regions. *λ*_1_, *λ*_2_ and *λ*_3_ are the linear coefficients of the predictor variables and *γ* is a scalar constant (intercept), which were determined on DORs and DDRs using a logistic regression model [22]. Context-dependent regions were not included in the parametrisation (Methods).

Definitions and detailed description of these terms are given in the S1 Text.

The *S*_*F*_*(R)* scoring function distinguishes between regions that undergo disorder-to-order and disorder-to-disorder transitions [22]. That is, increased local biases in the sequence composition as compared to the flanking regions facilitates ordering of the binding regions. The lack of such biases promotes formation of alternative contacts and a possible exchange between them, leading to disorder in the bound state and fuzzy (i.e. multimodal) interactions [22].

### Context-dependence of binding modes

To be able to perform sequence-based predictions at the single-residue level without additional information on the partner, we considered two problems: (1) the boundaries of an interacting protein region *R* are not known *a priori*, and (2) a given residue *A*_*i*_ in the region *R* can belong to interaction sites with different sizes and positions depending on the partner or cellular conditions {*R*_*i*_}.

**Fig 2:**
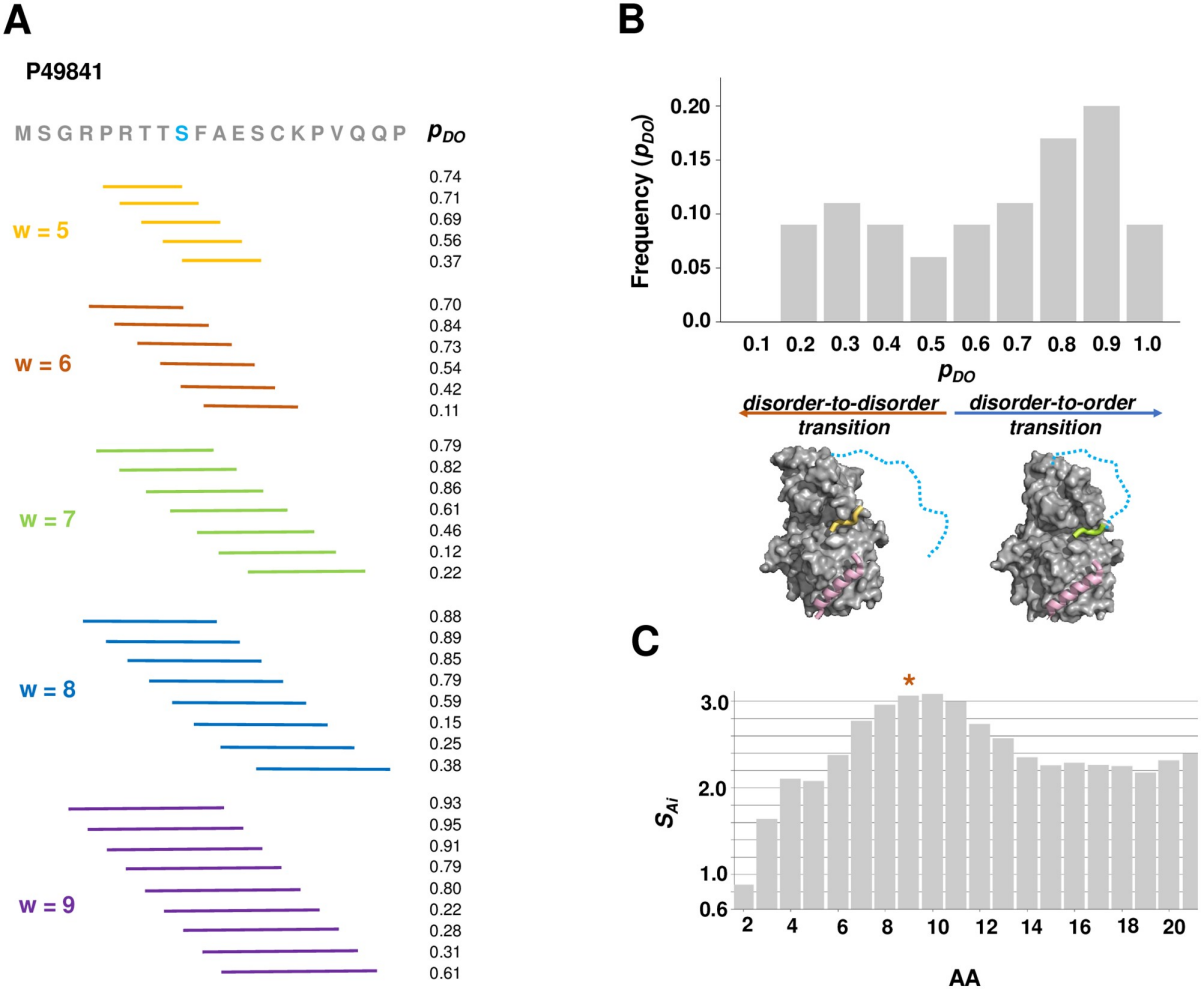
Determination of binding mode diversity. **(A) Assignment of possible binding sites.** The sequence of the N-terminal 20-residue region of glycogen synthase kinase 3 (GSK3, UniProt P49841) is shown. The possible 5 to 9 residue binding regions of Ser9 are displayed together with their probabilities for disorder-to-order transition (*p*_*DO*_*R*_*i*_). **(B) Frequencies of binding modes.** The distribution of the *p*_*DO*_*(R)* values for Ser9 are shown. The bimodal distribution of the *p*_*DO*_*(R)* values indicates that Ser9 can populate both disorder-to-order and disorder-to-disorder binding modes. The interactions with low-density lipoprotein receptor-related protein 6 (LRP6) peptides (wheat) and axin (violet) exemplifies the disorder-to-disorder binding modes (PDB: 4nm5), where the N-terminal region (dashed, cyan) does not adopt a well-defined structure in the complex. Phosphorylation of Ser9 induces folding of the N-terminal peptide (lime), which mediates an auto-inhibitory interaction (PDB: 4nm3)[18]. **(C) Shannon entropy for binding modes.** The Shannon entropy (Eq 5) is evaluated for the binding mode distribution of each residue. The $S_{A_{i}}$ values predict increased number of possible binding modes for residues 7–11, which is consistent with their conditional folding.

To solve these problems, we assigned a residue *A*_*i*_ to different possible binding regions (Fig 2), which represent interactions with different partners and conditions. Then we evaluated the *S*_*F*_*(R)* scoring function for each of these binding sites, which provided a distinct probability for disorder-to-order transition *p*_*DO*_*(R*_*i*_*)* for each of these hypothetical binding events (Methods). This procedure provided a set of *p*_*DO*_*(R*_*i*_*)* probabilities for all possible interacting regions of *A*_*i*_ (Fig 2). The probabilities for disorder-to-order and disorder-to-disorder transitions of *A*_*i*_ upon protein interactions can then be derived from such distributions as (see Methods).

$${p_{DO}(A_{i})=median\left\{p_{DO}(R_{i})\right\}}_{N}=median{\left\{\frac{exp\;S_{F}(R_{i})}{1+exp\;S_{F}(R_{i})}\right\}}_{N}$$ (3)

where *p*_*DO*_*(R*_*i*_*)* is the probability of disorder-to-order transition with a given binding site *R*_*i*_, *N* is the number of possible binding regions of *A*_*i*_ between a given length range (5–9 residues). The disorder-to-order transition probability of *A*_*i*_ is computed as the median of the distribution {*p*_*DO*_(*R*_*i*_)}_*N*_. The probability for disorder-to-disorder transition is obtained as *p*_*DD*_*(R*_*i*_) = 1-*p*_*DO*_*(R*_*i*_*)*. The FuzPred method predicts the *p*_*DO*_(*A*_*i*_) and *p*_*DD*_(*A*_*i*_) probabilities from the amino acid sequences, which characterize the most likely binding mode of residue *A*_*i*_ [22]. Earlier we had demonstrated that these residue-based *p*_*DO*_(*A*_*i*_) and *p*_*DD*_(*A*_*i*_) values can discriminate between residues belonging to different classes of binding modes (disorder-to-order, disorder-to-disorder and context-dependent) [22].

Here we address how the predicted binding mode of a given residue *A*_*i*_ varies with different binding sites. The distribution of {*p*_*DO*_(*R*_*i*_)}_*N*_ values (Eqs 2 and 3) characterizes the possible conformational transitions with a variety of partners, thus informs on the available binding modes. The frequency of a given binding mode, defined by the probability for disorder-to-order transition (*p*_*DO*_(*R*)) is given by

$$f[p_{DO}(R_{i})]=\frac{n_{R}[p_{DO}(R_{i})]}{N}$$ (4)

where *N* is the number of all possible binding sites around *A*_*i*_, and *n*_*R*_[*p*_*DO*_(*R*_*i*_)] is the number of binding regions with a binding mode *p*_*DO*_(*R*). To define *n*_*R*_[*p*_*DO*_(*R*_*i*_)] we have binned *p*_*DO*_(*R*) into 0.1 intervals.

Using the frequencies of all the possible binding modes of a given residue *A*_*i*_, we compute the Shannon entropy (Fig 2)

$$S_{A_{i}}=-\sum f[p_{DO}(R_{i})]log_{2}f[p_{DO}(R_{i})]$$ (5)

where *f*[*p*_*DO*_(*R*)] is the frequency of a given binding mode with a given *p*_*DO*_(*R*) (Eq 4). The sum runs over the bins of *p*_*DO*_(*R*).

Our approach is based on the assumption that the sequence-based prediction of the Shannon entropy (Eq 5) can quantify the diversity of binding modes of a given residue *A*_*i*_ (Fig 2) under many different cellular conditions and interaction partners, which are not known *a priori*. Low $S_{A_{i}}$ values reflect a strong preference for a given binding mode, whereas higher $S_{A_{i}}$ values indicate that different binding modes can be sampled under different conditions.

### The Shannon entropy discriminates context-dependent binding modes

We compared the Shannon entropy *$S_{A_{i}}$* of binding modes for all residues in the **DOR**, **DDR** and **CDR** datasets (Methods, S1 Table). We computed the *p*_*DO*_(*R*_*i*_) probabilities for each residue for all possible positions of binding sites in the 5–9 residue range using the full protein sequence (Eq 2) (Fig 2A). This process resulted in 35 predicted binding modes, in case all possible binding windows could be assigned (Methods). Fewer number of binding sites at the termini did not significantly affect the Shannon entropy values (S2 Fig). We divided the range of binding modes (*p*_*DO*_ [0,1]) into 10 bins, and determined the frequencies of the predicted binding modes for each residue in these 10 bins (Eq 4) (Fig 2B). The Shannon entropies of the possible binding modes were derived from such binding mode frequencies (Eq 5) (Fig 2C).

**Fig 3:**
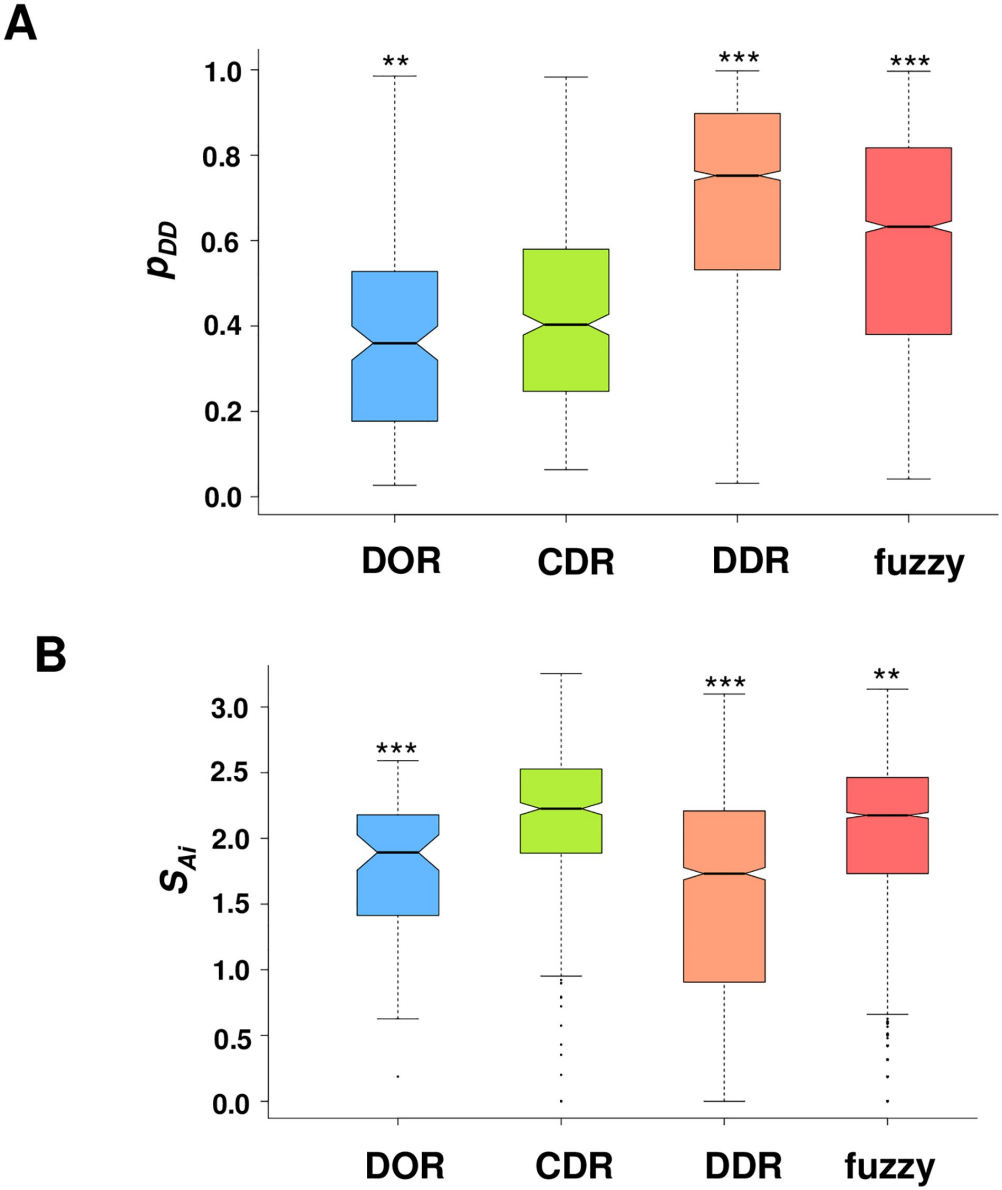
Predicted binding modes of disorder-to-order (DOR), context-dependent (CDR), disorder-to-disorder (DDR) and fuzzy regions. **(A) Binding mode probabilities.** The probabilities of disorder-to-disorder transitions are shown for DOR (blue), CDR (lime), DDR (salmon) and fuzzy (red) regions. The *p*_*DD*_ values indicate significantly elevated disorder for interactions of DDRs and fuzzy regions as compared to DORs and CDRs. **(B) Shannon entropy of binding modes. $S_{A_{i}}$** values for DOR (blue), CDR (lime), and DDR (salmon) regions significantly differ between these binding modes. Context-dependent regions exhibit the highest binding mode diversity as compared to DORs and DDRs. Fuzzy, disordered binding regions (from the Fuzzy Complexes Database [25]) also have elevated $S_{A_{i}}$ values indicating their context dependence. Statistical significances were determined by Mann-Whitney tests as implemented in the R program. p values as compared to CDRs are shown (** p < 10^−2^; *** p < 10^−5^).

The FuzPred predictions show that context-dependent regions exhibit more disordered interactions (higher *p*_*DD*_ values) than regions, which fold upon binding, while shifted towards more ordered interactions as compared to regions, which remain to be disordered in their complexes (Fig 3A). Context-dependent regions, however, exhibit the highest Shannon entropies as compared to **DOR** and **DDR** residues, which were observed in a unique binding mode (Fig 3B). The Shannon entropies (Eq 5) discriminate rather well between **DOR** and **CDR** datasets (AUC = 69.6%) as well as between **DDR** and **CDR** datasets (AUC = 72.0%) (Methods, S1 Table). $S_{A_{i}}$ values, however, do not differentiate between **DOR** and **DDR** datasets, which were observed in a uniform binding mode. Comparison of $S_{A_{i}}$ values of context-dependent, disorder-to-order and disorder-to-disorder regions mediating intra-molecular interactions corroborated that binding mode diversity discriminates between these binding modes [22] (S1 Fig).

We also compared these binding modes to fuzzy, disordered binding regions (**DBR**s), which exhibit multiple conformations when bound, with experimental evidence corroborating their contribution to binding affinity [25] (Methods, S1 Table). Fuzzy regions have comparable *p*_*DD*_ values to **DDR**s (Fig 3A), but have significantly higher $S_{A_{i}}$ values (Fig 3B). While **DBR**s are significantly more disordered in their bound states than **CDR**s (Fig 3A), the $S_{A_{i}}$ values of these binding modes are comparable (Fig 3B), indicating that fuzzy regions exhibit context-dependent binding modes, in accord with experimental data [25]. Taken together, these results suggest that the Shannon entropy values could be used to identify context-sensitive binding regions based on the diversity of interaction modes.

### FuzPred applications to predict context-dependent binding modes

We implemented the evaluation of Shannon entropy into the FuzPred method, which thus can estimate the pool of available binding modes from the sequence. Using both *p*_*DD*_(*A*_*i*_) and $S_{A_{i}}$ values, which are predicted by FuzPred, we can significantly discriminate context-dependent regions from disorder-to-order (**CDR** vs **DOR** AUC = 91.0%) and disorder-to-disorder regions (**CDR** vs **DDR** AUC = 93.6%).

In this section, we illustrate a range of applications of the FuzPred method by identifying context-dependent regions in different model systems.

#### Disordered binding regions

**Fig 4:**
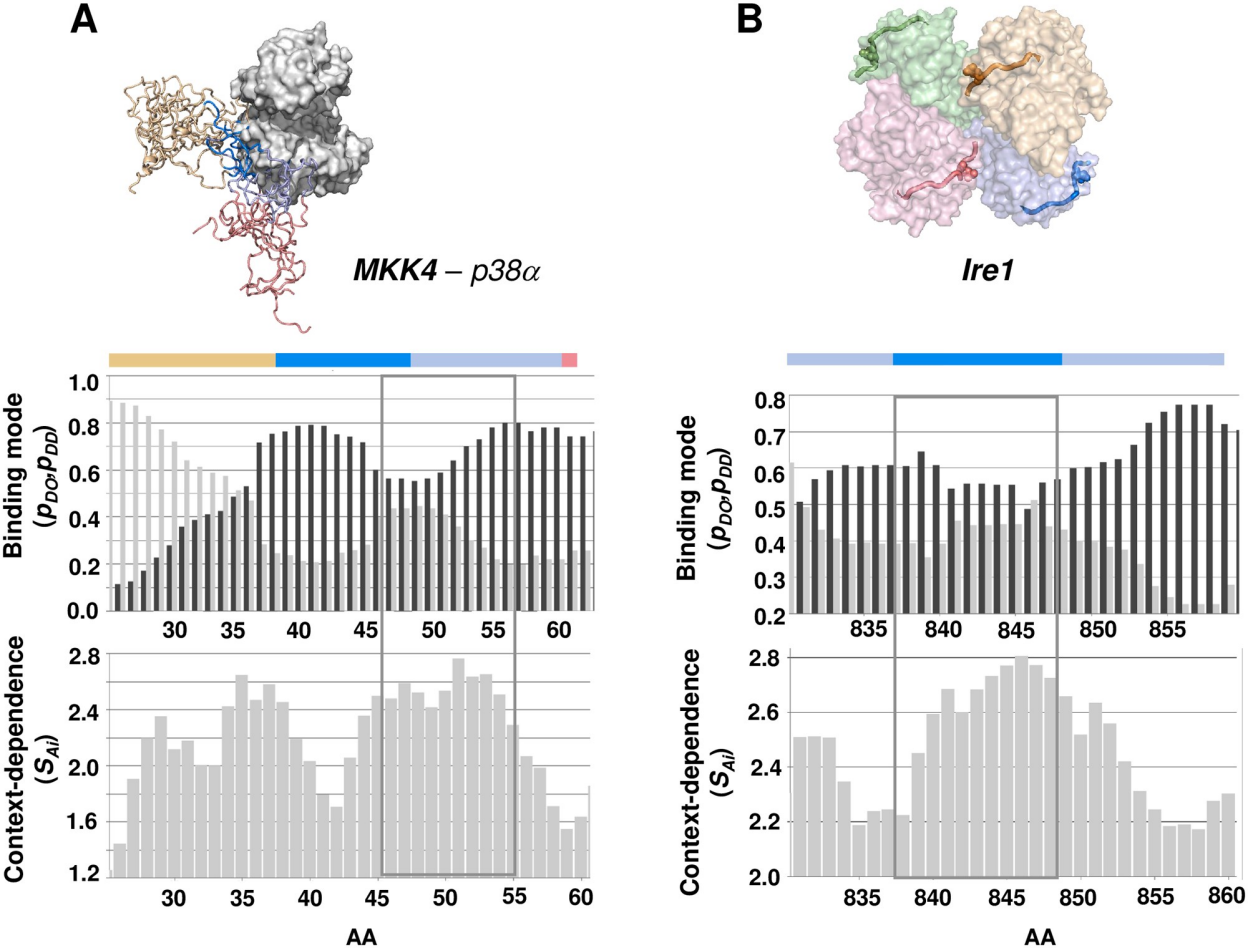
Prediction of context-dependent regions by the FuzPred method. **(A) Prediction of binding mode profiles.** Comparable probabilities for disorder-to-order transition (*p*_*DO*_, dark gray) and disorder-to-disorder transition (*p*_*DD*_, light gray) indicate a disordered binding mode for the region of residues 45–55 (grey box), which involves both the docking and the KIS motif, consistently with the experimental data [33] (*top panel*). Based on the binding profile, this region can fluctuate between ordered and disordered interactions (*bottom panel*), which will depend on the signaling pathway. The $S_{A_{i}}$ values indicate that both the docking motifs and the N-terminal part of the KIS domain are capable to establish different binding modes, consistent with their involvement in disordered interactions. Selected MKK4 conformers docked onto **p38α** structure (PDB:1lew). The docking motif (marine) and the KIS domain (light blue) are shown (coordinates as a courtesy of Dr. Malene Ringkjobing-Jensen). **(B) Prediction of phosphorylation-induced folding.** Trans-autophosphorylation induces folding of the activation loop in the dual-activity enzyme Ire1, which promotes its oligomerisation [19]. Packing of four monomers (wheat, light blue, pale green and light pink surfaces) (PDB: 3fbv) are stabilised by the ordered activation loop (cartoon, the phosphorylated Ser841 is shown by spheres). FuzPred predicts slightly higher probabilities for disorder-to-order transition (*p*_*DO*_, dark gray, *top panel*) for the activation loop (grey box) than for disorder-to-disorder transition (*p*_*DD*_, light gray, *top panel*), indicating that it can fold upon binding. The high $S_{A_{i}}$ values (*bottom panel*) corroborate that the activation loop can sample both disordered and ordered states in the bound form, which could be shifted towards the folded form by phosphorylation.

Mitogen activated protein kinase (MAPK) kinase MKK4 contacts its MAPK partner p38α via a canonical docking motif and a kinase specificity sequence (KIS). The canonical binding site has higher *p*_*DO*_ and low $S_{A_{i}}$ values indicating a more stable interaction site (Fig 4A). The 45-55-residue peptide has comparable *p*_*DO*_ and *p*_*DD*_ probabilities, indicating a possible variation of binding modes between ordered and disordered conformations (Fig 4A). The predicted increase in $S_{A_{i}}$ values corroborates the change in binding modes, leading to disordered binding. These results are in agreement with the calculated NMR transverse relaxation rates (R_2,bound_), which reflect sizeable conformational fluctuations in the MKK4-p38α complex (Fig 4A) [33]. As the bound structures of the docking motif are similar with different partners, variable binding modes of the KIS domain are important to tune specificity for p38α [33].

#### Phosphorylation-induced folding

Folding as well can be induced by post-translational modifications, which may interfere with binding. For example, inositol-requiring enzyme 1 (Ire1) conveys unfolded protein response signals via oligomerization, which activates both its kinase and RNase domains [19]. Ire1 trans-autophosphorylation triggers a disorder-to-order transition of the activation loop, which in turn provides a positive feedback for oligomer assembly. In agreement with these observations, residues 836–848 exhibit elevated $S_{A_{i}}$ values indicating a possible change in binding mode upon phosphorylation (Fig 4B). The predicted comparable *p*_*DO*_ and *p*_*DD*_ values further support changes in binding modes (Fig 4B).

#### Transient binding sites

The nonsense-mediated decay factor regulator of nonsense transcripts 2 (UPF2) binds its partner regulator of nonsense transcripts 1 (UPF1) in a bi-partite manner. The linker (1130-1166-residue), which connects the structured binding elements however, remains disordered in the bound state [34], yet contributes substantially to the binding affinity of UPF2. FuzPred predicts elevated $S_{A_{i}}$ values in particular in the middle of the linker, indicating a variation in binding modes (S3 Fig). This finding is in accord with the increased probability for disorder-to-order transition, indicating transient interactions of the linker via conditional folding (S3 Fig).

### Binding mode landscapes

**Fig 5:**
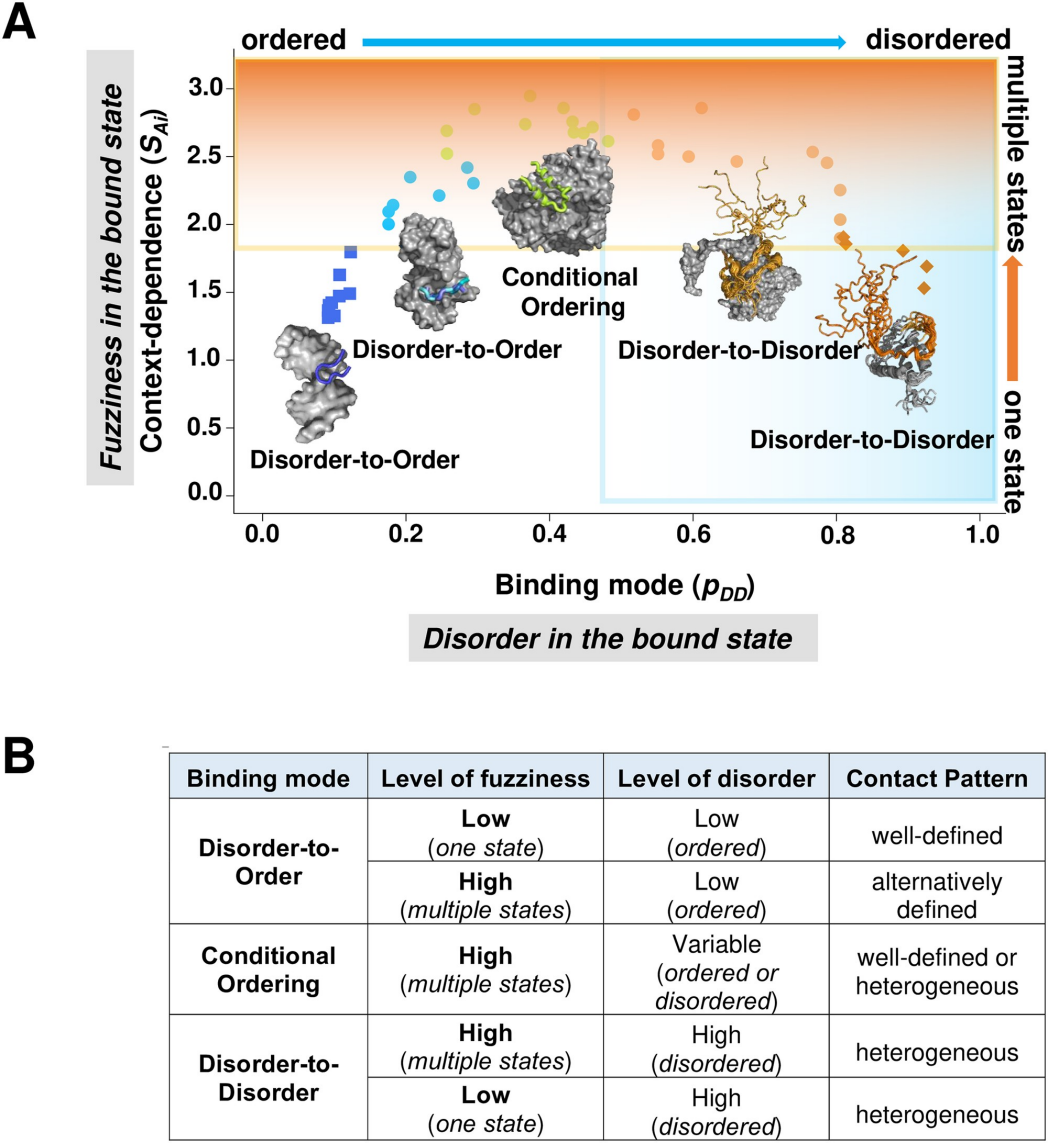
A binding mode landscape for disordered protein interactions. Residues are characterised by their binding modes to increase or decrease order upon interactions and the context-dependence of such binding modes. (**A**) The binding modes, reflecting the level of disorder in the bound state, are represented on the x axis; ranging from structured, well-defined to disordered, heterogeneous interactions, as quantified by the *p*_*DD*_ values. Context-dependence, reflecting the level of fuzziness, is displayed on the y axis, ranging from stable, uniform to diverse, inducible binding modes, as quantified by the $S_{A_{i}}$ values. The *p*_*DD*_ and *$S_{A_{i}}$* values are predicted from the sequence by the FuzPred program. A **disorder-to-order** binding with low context-dependence is exemplified by a disordered loop (504–512 aa, *blue squares*) in Taq polymerase, which adopts a stable structure upon interacting with DNA (PDB: 3lwl [54]). A **disorder-to-disorder** binding with low context-dependence is represented by the heterogeneous interactions between the elongation factor AF4 (residues 747–754, *orange diamonds*) with leukemia fusion protein AF9 (PDB:2lm0 [55]). **Fuzzy, context-depedent interactions** sample a wide variety of binding modes ranging from disorder-to-order to disorder-to-disorder transitions. Context-dependent **disorder-to-order** binding is exemplified by the **polymorphic interactions** of the ribosomal S6 kinase 1 (RSK1, residues 697–703, *light blue dots*), which adopts different secondary structures upon binding to S100B, corresponding to autoinhibited and active forms (PDB:5csf, 5csi, 5csj [23]). **Conditional folding** upon binding is represented by the N-terminal region (residues 15–25, *lime dots*) of the large chain of ribonucleoside-diphosphate reductase, which can be structured or disordered in different oligomers (PDB: 1zyz, 1zzd [56]). Context-dependent **disordered binding** is exemplified by the p150 subunit of the eukaryotic initiation factor 4F (residues 225–235, *light orange dots*). eIF4 wraps around the translation initiation factor 4E, but the flanking region remains to be highly dynamic in the assembly (PDB: 1rf8 [57]). The interaction sites are shown by the same colours as interaction modes, and partner proteins are displayed by grey surfaces. **(B)** The characteristics of the different binding modes, which are represented in panel **A**. The binding mode landscape comprises a continuum of interaction behaviours, the major trends of which are illustrated by the distinct modes.

The *p*_*DD*_ and $S_{A_{i}}$ values define a two-dimensional landscape for context-dependent protein interactions (Fig 5). Such binding mode landscape characterises the extent to which residues undergo disorder-to-order or disorder-to-disorder transitions upon binding, and the strength of their preference for such binding modes or context-dependence. The x axis defines the level of disorder in the bound state, ranging from structured, well-defined to disordered, heterogeneous interactions, as quantified by the *p*_*DD*_ values; whereas the y axis defines the number of binding modes, or fuzziness (Fig 5), as quantified by the $S_{A_{i}}$ values. Points at the bottom of the landscape represent transitions with low level of context-dependence and one bound state, while points at the upper part of the landscape represent context-dependent transitions with multiple bound states. The binding mode landscape represents a continuum of interaction scenarios, out of which we discuss some distinct modes below.

Points on the bottom left of the landscape (*p*_*DD*_ < 0.25 and $S_{A_{i}}$ < 1.8) have a strong preference for disorder-to-order transitions, and fold into a stable structure in the bound complex (Fig 5). DORs establish well-defined interactions with the partner and are visible in the electron density of complex crystal structures.

By contrast, residues at the bottom right of the landscape (0.65 < *p*_*DD*_ and $S_{A_{i}}$ < 1.8) tend to increase their flexibility or unfold in the bound states (Fig 5). DDRs exhibit highly heterogeneous conformations, and many redundant interaction patterns, detailed structural characterisation of which presents a challenge for most experimental methods. DDRs have a strong preference to remain disordered in the bound states, so cellular conditions unlikely trigger their disorder-to-order transitions.

In the upper region of the landscape, residues exhibit a variety of binding modes with different partners or cellular conditions (Fig 5). Context-dependent regions include: (1) polymorphic regions (*p*_*DD*_ ≤ 0.25 and 2.25 < $S_{A_{i}}$), which fold into alternative structures with different partners, (2) conditionally folding regions (0.25 < *p*_*DD*_ ≤ 0.45 and 2.25 < $S_{A_{i}}$), which can be induced into a well-defined structure by specific partners or post-translational modifications, and (3) disordered binding regions (0.45 < *p*_*DD*_ ≤ 0.75 and 2.25 < $S_{A_{i}}$), which exhibit conformational exchange in the complex (Fig 5). All these context-dependent regions are fuzzy [12], as they can exhibit a wide variety of binding modes.

We also observe that the top left and right corners of the landscape have no data points showing that residues with strong probabilities for DO or DD transitions unlikely visit other binding modes. In contrast, residues with the *p*_*DD*_ ~ 0.2–0.8 are prone to changing their binding modes, and are unlikely sample the same type of interaction under different conditions, leading to paucity of data in the bottom middle of the landscape (Fig 5).

**Fig 6:**
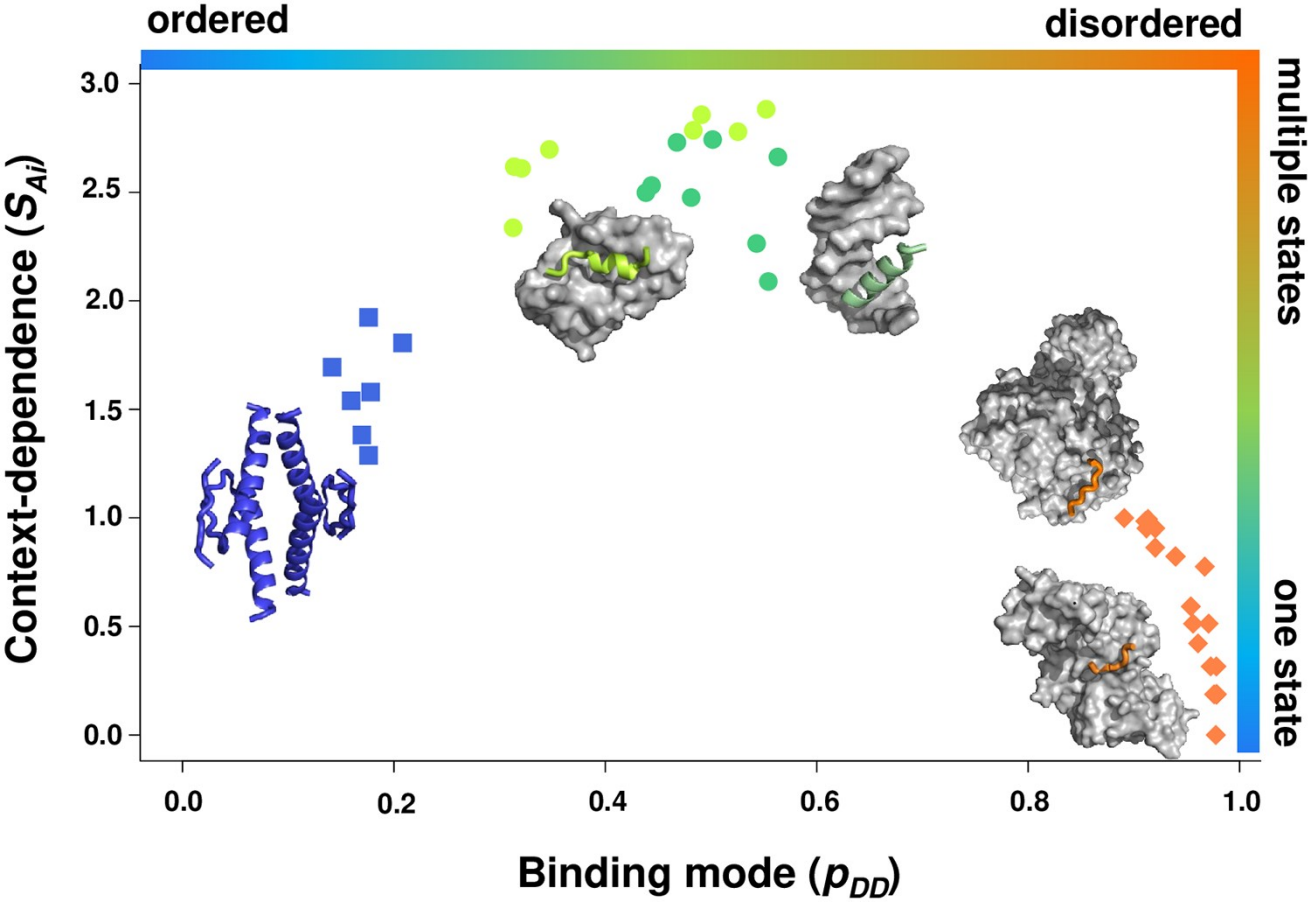
Binding mode landscape for p53 interactions. The oligomerisation domain (residues 325–356, blue squares) exhibits a strong preference for disorder-to-order transitions and forms stable tetramers (PDB:1c26) [36] and higher-order structures. Short linear peptides (residues 378–386, orange diamonds) at the disordered C-terminal regulatory region interact with sirtuin (PDB: 4zzj [37]) and the cyclin dependent kinase cyclin A (PDB:1h26 [38]) exhibit heterogeneous binding modes. On the top of the binding mode landscape two context-sensitive regions are shown. The disordered N-terminal transactivation region interacts with Mdm2 (PDB:1ycr [58]) via a short helical segment (19–25 aa, lime dots). The beginning of the disordered C-terminal region folds into an α-helical conformation (residues 278–285, green dots) to recognise DNA via a variety of dynamic binding modes (PDB: 2ady, [36]). The high $S_{A_{i}}$ values for both regions indicate fuzzy interactions, which are strongly influenced by the cellular context. The interaction sites are shown by the same colours as interaction modes, and partner proteins are displayed by grey surfaces.

We illustrate the type of insights that can be obtained from the analysis of the binding mode landscape by considering the case of the tumor suppressor p53 (Fig 6). p53 is an interaction hub, which binds to multiple partners in a variety of cellular processes. The N- and C-terminal regions of p53 are disordered, and comprise many linear interaction motifs [35]. FuzPred predictions indicate that these interactions sample a wide variety of different binding modes. These calculations indicate a strong preference for a disorder-to-order transition for the oligomerisation domain (residues 325–356, *bottom left on the landscape*, Fig 6), which forms stable tetramers (PDB:1c26) [36] and can be involved in higher-order structures. In contrast, the C-terminal region of p53 is predicted to remain disordered in the bound state, without considerable ordering of the binding sites. This result is in agreement with the observation that the C-terminal regulatory region of p53 interacts with sirtuin [37] and the cyclin-dependent kinase cyclin A [38] through short disordered peptide motifs (residues 378–386, *bottom right on the landscape*, Fig 6). The *p*_*DD*_ and $S_{A_{i}}$ values of the motif in the p53 N-terminal transactivation domain that is responsible for the binding of mouse double minute 2 (Mdm2) (residues 19–26, *top*, *middle of the landscape*) indicate a large variability of binding modes. Indeed, this segment is also engaged in interactions with the high mobility group box 1 (HMGB1) protein [39] and the transcriptional co-activators CREB-binding protein (CBP) and its homolog p300 [40]. The DNA recognition helix (residues 278–285, *top*, *middle of the landscape*, Fig 6) is predicted to have variable binding modes, which may be responsible for differential DNA recognition [36].

## Discussion

It is increasingly recognized that a finely-tuned regulation of cellular pathways is enabled by a wide variety of protein binding modes. Such binding modes involve a range of conformational transition, from folding (ordering) to unfolding (disordering), and may vary with different partners, cellular conditions or be modulated by post-translational modifications. In many cases, protein regions sample different binding modes and alternate between structured and disordered states in the bound forms. Previously, we had demonstrated that the continuum of binding modes, the extent to which proteins undergo disorder-to-order transitions or remain disordered, can be predicted from the sequence without specifying the binding partners [22].

Here we have asked how the context-dependent binding of proteins is encoded in their amino acid sequences, and whether it is possible to predict the multiplicity of their possible binding modes. We have shown that this goal can be achieved by defining the Shannon entropy associated with the probabilities of the binding modes predicted by the FuzPred method.

We have then discussed how the analysis of the binding modes and of their context-dependence defines a binding mode landscape, which represents a continuum of interaction behaviours. The binding mode landscape shows how interactions can change with cellular conditions, out of which we analysed a few distinct modes. The left and right sides of this landscape includes residues that are likely to adopt a specific interaction mode with many partners. By contrast, in the top region of the landscape, high entropy values indicate a variety of context-dependent binding modes.

Taken together, the results that we have reported illustrate how the FuzPred algorithm can contribute to the current efforts to predict the binding behaviour of disordered proteins from their amino acid sequences, without prior information on their partners. We anticipate that our approach will facilitate the study of polymorphic, conditionally folding and disordered binding regions, which sample a wide range of different binding modes that can be influenced by the cellular conditions. These fuzzy regions often serve as regulatory switches in a variety of cellular processes [41] and shift their binding modes upon post-translational modifications [42], allosteric effects [43] or higher-order organisation [15, 19]. Context-dependent binding modes impart functional variability on linear motifs, which are involved in multiple pathways [44, 45]. Finally, predicting inducible interaction sites from sequences may also help identify sites for small molecule interactions [46, 47].

## Methods

### Datasets

#### Regions representing disorder-to-order binding mode (DORs, S1 Table)

Disordered regions (≥ 5 AA) in monomeric proteins, defined as residues with missing coordinates in the PDB were collected in crystal structures with resolution higher than 3 Å. Protein sequences with post-translational modifications or non-standard amino acids were excluded. Structures were also analysed for truncation artefacts. Sequence with >75% similarity were excluded using the CD-hit program [48]. We then collected all available complex structures of disordered regions with the same sequence by projecting them to their UniProt reference. In each crystal structure, we assigned order or disorder for all residues of the disordered regions (Fig 1). In case at least 5 consecutive residues were observed with a well-defined conformation in all complexes of the disordered regions were defined as DORs. In the DOR dataset, we only included those sequences, where at least 1 residue mediated inter-molecular interaction (within 4.5 Å from the interface). Homotypic interactions (dimerisation, oligomerisation) were also considered as inter-molecular contacts. The **DOR** dataset contained 97 disordered regions, which were represented in 331 complexes (535 chains) only in a disorder-to-order binding mode.

#### Regions representing context-dependent binding modes (CDRs, S1 Table)

Disordered regions, which were structured or remained undetected in different complexes were assembled in the **CDR** dataset. In case at least 5 consecutive residues were observed in more, than one binding mode, and at least 1 residue mediated inter-molecular contacts in the ordered form it was defined as a context-dependent region (CDR, Fig 1). The **CDR** dataset contained 96 disordered regions, with alternative binding modes in 750 complex structures (1505 chains) (S1 Table).

#### Regions representing disorder-to-disorder transitions (DDRs, S1 Table)

DDRs were assembled from the PDB. We considered those regions, which were missing from both the monomeric and the complex forms. We collected 338 regions with disorder-to-disorder binding modes representing 583 complexes (1419 chains) (S1 Table).

#### Regions representing fuzzy, disordered binding regions (DBRs, S1 Table)

Regions that exhibit conformational exchange in their bound states were assembled from the Fuzzy Complexes Database v3.3 (http://protdyn-database.org) [25]. Out of the 92 disordered complexes in FuzDB (evidenced by a range of experimental methods), we selected 56 regions, where PDB structures of the complexes were available (S1 Table).

### Quantifying binding modes

#### Computing *p*_*DO*_ and *p*_*DD*_ values for regions

Binding modes were characterised based on whether protein regions tend to increase (*p*_*DO*_) or decrease order (*p*_*DD*_) upon interactions. The simultaneous determination of the *p*_*DO*_ and *p*_*DD*_ probabilities provides a continuous scale for the binding modes. To evaluate *p*_*DO*_(*R*) and *p*_*DD*_(*R*), the scoring function (Eq 2) was computed for selected regions, based on the local bias in disorder [31], amino acid composition and Kyte-Doolittle hydrophobicity [49] (S1 Text). Parameters of the scoring function were trained to distinguish between disorder-to-order and disorder-to-disorder regions, but not including context-dependent regions. The scoring function was evaluated in running windows ranging from 5 to 9 residues around each residue, using the full protein sequence (Fig 2). These windows represented the possible interaction sites, the length of the which was based on our earlier analysis of disorder-to-order binding regions [22]. *S*_*F*_*(R*_*i*_*)* was computed for each of these sites (Eq 2, Extended methods) and *p*_*DO*_*(R*_*i*_*)* was determined accordingly (Eq 1).

#### Distributions the *p*_*DO*_ and *p*_*DD*_ values with different binding sites

Using running windows from 5 to 9 residues provide 35 *p*_*DO*_*(R*_*i*_*)* values in case all the possible binding sites could be defined. The distribution of the *p*_*DO*_*(R*_*i*_*)* values were computed in 10 bins between [0,1], representing the whole spectrum of binding modes (Eq 4). The modality and width of the {*p*_*DO*_(*R*_*i*_)}_*N*_ distribution informs on the number and preference of binding modes.

#### Shannon entropy of binding modes

The Shannon entropy associated with the {*p*_*DO*_(*R*_*i*_)}_*N*_ distribution was calculated for each residue using frequencies of *p*_*DO*_*(R*_*i*_*)* values. Thus, the $S_{A_{i}}$ Shannon-entropy, similarly to the most likely binding mode *p*_*DO*_*(A*_*i*_*)* characterizes interactions of a residue. Low $S_{A_{i}}$ values reflect a preference for a distinguished binding mode, whereas higher values indicate that the given residue can sample multiple binding modes under different conditions. *p*_*DO*_*(A*_*i*_*)* and $S_{A_{i}}$ inform whether a given residue tends to be more or less ordered upon binding and to what extent this binding mode can be modulated by the environment. The values of the Shannon entropy depend on the number of bins used for the *p*_*DO*_*(R*_*i*_*)* distribution. Using more bins (> 10) would require defining more binding sites, including longer interfaces. This is, however, not typical for disordered proteins [50, 51] and would decrease the local bias of the binding motifs.

We also eliminated potential artefacts owing the reduced number of hypothetical binding sites at the N- and C-terminal regions as compared to the middle of the sequence (S2 Fig). We did not find a significant difference between the Shannon entropies of the 10-residue long N- and C- terminal regions as compared to 10 aa regions in the middle of the sequence analysing 2000 randomly selected human proteins (S2 Fig). At the same time, disorder predictions exhibit strong differences between terminal and inner segments owing to the asymmetric environment (S2 Fig).

#### Evaluation of performance

Receiver operating characteristic (ROC) curves were computed using the R program. The true positive rate (TPR) was calculated as a function of the false positive rate (FPR, sensitivity) using the experimentally observed disorder-to-order, disorder-to-disorder and context-dependent regions. The area-under-the-curve (AUC) was determined by the R program. Only disordered residues were included in the distinct binding mode classes.

## Supporting information

S1 Text Extended methods, description of the scoring function. (DOCX)

S2 Text Supporting references. (DOCX)

S1 Table datasets for disorder-to-order, context-dependent, disorder-to-disorder and fuzzy interactions. (XLSX)

S1 Fig Predicted binding modes for intrinsically disordered regions, mediating intra-molecular interactions. (DOCX)

S2 Fig Predicted disorder, binding modes and context-dependence of N- and C-terminal regions as compared to the middle of the sequence. (DOCX)

S3 Fig Predicted binding modes and context-dependence for transient binding sites in the UPF2-UPF1 complex. (DOCX)
