# Supplementary material for: Sequence-based prediction of protein binding mode landscapes
Source: PLoS Comput Biol. 2020 May 26;16(5):e1007864. doi: 10.1371/journal.pcbi.1007864 (PMC7304629; doi:10.1371/journal.pcbi.1007864)
Supplement: S1 Text — (DOCX) [file pcbi.1007864.s001.docx]

**Supplementary information**

**S1 Text**

**Extended methods**

**The scoring function**

The scoring function was expressed as a linear combination of three terms, which express the local bias of *R* in disorder, composition and dynamics as compared to the flanking regions (Eq. 2, main text). Below we define the individual terms of the scoring function.

**Local dynamics bias**

We propose a general scheme based on the difference between the average degree of disorder of the region of interest and its flanking regions. Here we used the Espritz NMR method [1] to compute the disorder scores, but we have shown previously that the approach also works with other disorder prediction algorithms [2]. More detailed discussion on calculation of disorder can be found in the main text (section '**Probabilities of the binding modes of disordered regions'**) as well as in our previous study [2].

We determined the average disorder score for the region of interest *R*

$${ID}_{R}=\frac{\sum_{i=R_{start}}^{R_{end}} {Sc}_{ID,i}}{L_{R}} (1)$$

where $R_{start}$and $R_{end}$ indicate the first and the last residue, respectively, of *R*. ${Sc}_{ID,i}$ is the disorder score of residue *i* within *R*; these disordered scores are summed for all the residues in R, and the result is then divided by the length $L_{R}$ of R.

We have also computed the average disorder score of the flanking regions:

$${ID}_{FlN}=\frac{\sum_{j=R_{start}-w_{Fl,ID}}^{R_{start}-1} {Sc}_{ID,j}}{w_{Fl, ID}} (2)$$

$${ID}_{FlC}=\frac{\sum_{j=R_{end}+1}^{R_{end}+w_{Fl,ID}} {Sc}_{ID,j}}{w_{Fl, ID}} (3)$$

where ${ID}_{avg, FlN}$ and${ID}_{avg, FlC}$ are the average disordered scores of the N- and C- flanking region, respectively, and $w_{Fl, ID}$ is the flanking window size ($w_{Fl, ID}=15$ residues). If *R* is closer than $w_{Fl, ID}$to either terminus, only one of the flanking regions can be used.

Then we have determined the difference in disorder preference between R and its flanking regions:

$${\Delta ID}_{R,FlN}={abs(ID}_{R} -{ID}_{FlN}) (4)$$

$${\Delta ID}_{R,FlC}={abs (ID}_{R} -{ID}_{RFlC}) (5)$$

The larger of the two values (Eqs. 4-5) was selected:

${\Delta ID}_{R,Fl}=max({\Delta ID}_{FlN}; {\Delta ID}_{FlC})$ (6)

${\Delta ID}_{R,Fl}$ given in Eq. (6) estimates the bias in dynamics of *R* as compared to its local environment.

**Compositional bias**

We have determined the propensity of each of the 20 amino acid types in *R*

$$A_{i,R}=\frac{N_{i,R}}{L_{R}} (7)$$

where $N_{i,R}$ is the number of amino acid type *i* in the region *R*.

We have determined the propensity of each of the 20 amino acid types for the N- and C- flanking regions of *R*:

$$A_{i,FlN}= \frac{N_{i,FlN}}{w_{Fl, A}} (8)$$

$$A_{i,FlC}= \frac{N_{i,FlC}}{w_{Fl, A}} (9)$$

where $A_{i,FlN}$ and $A_{i,FlC}$ is the propensity of amino acid type *i* in the N- and C-flanking region and $w_{Fl, A}$ is the flanking window size ($w_{Fl, A}=20$ residues)

We computed the difference in propensity of each amino acid between *R* and its N- or C-flanking regions:

$\Delta A_{i,FlN}=abs(A_{i,R}-A_{i,FlN})$ (10)

$\Delta A_{i,FlC}=abs(A_{i,R}-A_{i,FlC})$ (11)

The total compositional difference was computed by summarizing the differences in amino acid propensities (Eqs. 10, 11) for the 20 amino acid types:

$${\Delta A}_{R,FlN}=(\sum_{i=1}^{20} {\Delta A}_{i,FlN})/2 (12)$$

$${\Delta A}_{R,FlC}=(\sum_{i=1}^{20} {\Delta A}_{i,FlC})/2 (13)$$

where ${\Delta A}_{R,FlN}$ and ${\Delta A}_{R,FlC}$ is the difference in composition between *R* and its N- and C-flanking region. The division by 2 was applied to normalize between 0 and 1.

The larger out of the ${\Delta A}_{R,FlN}$ and ${\Delta A}_{R,FlC}$ values (Eqs. 12, 13) was used to estimate compositional bias of *R* as compared to its flanking regions:

${\Delta A}_{R,Fl}=\max\left( {\Delta A}_{R,FlN} ,{\Delta A}_{R,FlC} \right) (14)$

The compositional term given in (Eq. 14) expresses the enrichment/depletion of some amino acids in *R*, but does not depend on their actual types.

**Hydropathy bias**

The average hydrophobicity of *R* was computed based on the Kyte-Doolittle scale[3]:

$$H_{R}=\frac{\sum_{i=R_{start}}^{R_{end}} H_{i}}{L_{R}} (15)$$

where *H_i_* is the hydrophobicity index of each residue and *L_R_* is the length of the region of interest.

We computed the average hydrophobicity of *R* and the N- or C-flanking regions:

$$H_{FlN}=\frac{\sum_{i=R_{start}-w_{Fl,H}}^{R_{start}-1} H_{i}}{w_{Fl, H}} (16)$$

$$H_{FlC}=\frac{\sum_{i=R_{end}+1}^{R_{end}+w_{Fl,H}} H_{i}}{w_{Fl, H}} \left( 17 \right)$$

where $H_{FlN}$ and $H_{FlC}$is the average hydrophobicity of the N- and C- flanking region, and $w_{Fl, H}$ is the flanking window size ( $w_{Fl, H}=20)$.

Difference in hydrophobicity between the *R* and its flanking regions was obtained as:

$$\Delta h_{R,FlN}=(H_{R}-H_{FlN}) (18)$$

$$\Delta h_{R,FlC}=\left( H_{R}-H_{FlC} \right) (19)$$

where $\Delta h_{R,FlN}$ and $\Delta h_{R,FlC}$ is the difference in hydrophobicity between *R* and its N- and C- flanking region.

The difference in hydrophobicity (Eqs. 18, 19) was then weighted by the difference in composition (Eqs. 12, 13), assuming that hydrophobicity only matters for residues with a strong compositional bias:

$$\Delta H_{R,FlN}={\Delta A}_{R,FlN} *\Delta h_{R,FlN} (20)$$

$$\Delta H_{R,FlC}={\Delta A}_{R,FlC} *\Delta h_{R,FlC} (21)$$

where ${\Delta A}_{R,FlN}$ and ${\Delta A}_{R,FlC}$ is the difference in composition between *R* and its N- and C- terminal flanking region (Eqs. 12, 13).

The hydropathy bias was defined by the larger of the $\Delta H_{R,FlN}$, $\Delta H_{R,FlC}$ values:

$${\Delta H}_{R,Fl}=\max\left( {\Delta H}_{R,FlN} ,{\Delta H}_{R,FlC} \right) (22)$$

where ${\Delta H}_{R,Fl}$expresses the bias in hydropathy of *R* as compared to its flanking regions.

**Flanking window size**

We varied the flanking window size to compute ${\Delta ID}_{R,Fl}, {\Delta A}_{R,Fl}$, ${\Delta H}_{R,Fl}$biases between 5 and 30 residues [2] and found a significant deviation between these terms for DORs and DDRs, respectively. We used $w_{Fl, ID}=15$ residues for ${\Delta ID}_{R,Fl},$, and ${w_{Fl, A}=w}_{Fl, H}=20$ residues to evaluate ${\Delta A}_{R,Fl}, {\Delta H}_{R,Fl}$ as they gave the largest discrimination between DOR and DDR classes. If the length of the disordered region exceeded 30 residues, the flanking window is defined on the boundary of the disordered region.

**Parameters of the scoring function**

The linear coefficients of the scoring function (Eq. 2, main text) were defined using a binary logistic model [2]. The probability *p_DO_(R)* expressed our degree of confidence in assigning a region *R* to the DOR class, and *p_DD_(R)*=1- *p_DO_(R)*, correspondingly, to the DDR class. Here we used the original parameters, the training of which was described in [2]. Importantly, context-dependent residues were not involved in the parametrisation.
