# Supplementary material for: Sequence-based prediction of protein binding mode landscapes
Source: PLoS Comput Biol. 2020 May 26;16(5):e1007864. doi: 10.1371/journal.pcbi.1007864 (PMC7304629; doi:10.1371/journal.pcbi.1007864)
Supplement: S2 Text — (DOCX) [file pcbi.1007864.s002.docx]

**S2 Text**

**References**

1. Walsh I, Martin AJ, Di Domenico T, Tosatto SC. ESpritz: accurate and fast prediction of protein disorder. Bioinformatics. 2012;28(4):503-9. Epub 2011/12/23. doi: 10.1093/bioinformatics/btr682. PubMed PMID: 22190692.

2. Miskei M, Horvath A, Vendruscolo M, Fuxreiter M. Sequence-Based Prediction of Fuzzy Protein Interactions. J Mol Biol. 2020;in press. doi: <https://doi.org/10.1016/j.jmb.2020.02.017>.

3. Kyte J, Doolittle RF. A simple method for displaying the hydropathic character of a protein. J Mol Biol. 1982;157(1):105-32. Epub 1982/05/05. PubMed PMID: 7108955.

4. Miskei M, Antal C, Fuxreiter M. FuzDB: database of fuzzy complexes, a tool to develop stochastic structure-function relationships for protein complexes and higher-order assemblies. Nucleic Acids Res. 2017;45(D1):D228-D35. doi: 10.1093/nar/gkw1019. PubMed PMID: 27794553; PubMed Central PMCID: PMC5210525.

5. Clerici M, Mourao A, Gutsche I, Gehring NH, Hentze MW, Kulozik A, et al. Unusual bipartite mode of interaction between the nonsense-mediated decay factors, UPF1 and UPF2. EMBO J. 2009;28(15):2293-306. Epub 2009/06/27. doi: emboj2009175 [pii]

10.1038/emboj.2009.175. PubMed PMID: 19556969; PubMed Central PMCID: PMC2726699.
