# Supplementary material for: Sequence-based prediction of protein binding mode landscapes
Source: PLoS Comput Biol. 2020 May 26;16(5):e1007864. doi: 10.1371/journal.pcbi.1007864 (PMC7304629; doi:10.1371/journal.pcbi.1007864)
Supplement: S1 Fig — (DOCX) [file pcbi.1007864.s004.docx]

**S1 Figure Predicted binding modes (A) and context-dependence (B) of disorder-to-order (DOR), context-dependent (CDR) and disorder-to-disorder (DDR) regions, mediating inter- and intramolecular interactions. (A) Binding modes.** The probabilities of disorder-to-disorder transitions are shown for DOR (blue), CDR (lime) and DDR (salmon) regions. The *p_DD_(A_i_)* values indicate significantly elevated dynamics for interactions of DDRs as compared to DORs and CDRs. **(B) Context-dependence.** The Shannon entropy of binding modes **(***S(A_i_)* values) for DOR (blue), CDR (lime) and DDR (salmon) regions significantly differ between these binding modes. Context-dependent regions exhibit the highest binding mode diversity as compared to DORs and DDRs. Datasets are from Table S1-S3 of reference [2]. Statistical significance was determined by Mann-Whitney tests as implemented in the R program. p values as compared to CDRs are shown (*** p < 10^-5^).
