# Supplementary material for: Sequence-based prediction of protein binding mode landscapes
Source: PLoS Comput Biol. 2020 May 26;16(5):e1007864. doi: 10.1371/journal.pcbi.1007864 (PMC7304629; doi:10.1371/journal.pcbi.1007864)
Supplement: S2 Fig — (DOCX) [file pcbi.1007864.s005.docx]

**S2 Figure Predicted disorder (A), binding modes (B) and context-dependence (C) of N- and C-terminal regions as compared to the middle of the sequence.** Disorder scores by Espritz NMR [1], *p_DD_* values by FuzPred [2] and Shannon-entropy values ($S_{A_{i}}$ ) were averaged for 10 residues in the N- (**N**, *light blue*) and C-terminal (**C**, *dark blue*) regions, as well as for a 10-residue segment, which was defined in the middle of the sequence (**M**, *lime*). Statistical significance was determined by Mann-Whitney tests as implemented in the R program. p values as compared to the middle of the sequence are shown (*** p < 10^-5^). While disorder in free and bound state considerably varies between the ends and the inner part of the sequence, the Shannon entropies do not exhibit significant difference between these regions.
