# Supplementary material for: Sequence-based prediction of protein binding mode landscapes
Source: PLoS Comput Biol. 2020 May 26;16(5):e1007864. doi: 10.1371/journal.pcbi.1007864 (PMC7304629; doi:10.1371/journal.pcbi.1007864)
Supplement: S3 Fig — (DOCX) [file pcbi.1007864.s006.docx]

**S3 Figure Predicted binding modes (A) and context-dependence (B) for transient binding sites in the UPF2-UPF1 complex (C).** The nonsense mediated decay factor UPF2 binds its partner UPF1 (grey) via two structured binding elements (*lime*), which are connected by a linker (residues 1130-1166, magenta, dotted line), which is invisible in the bound complex (PDB:2vjw [5]). FuzPred predicts elevated $S_{A_{i}}$ values in particular in the middle of the linker (gray box), indicating that it is capable to establish more stable interactions. Transient binding of the linker is consistent with its contribution to binding affinity [5]. Conditional folding of the linker is also supported by the elevated probabilities for disorder-to-order transition for the region of residues 1145-1153 (*p_DO_*, dark gray).
